# Roles of RNA m6A modification in nonalcoholic fatty liver disease

**DOI:** 10.1097/HC9.0000000000000046

**Published:** 2023-01-27

**Authors:** Jian Tan, Yue-fan Wang, Zhi-hui Dai, Hao-zan Yin, Chen-yang Mu, Si-jie Wang, Fu Yang

**Affiliations:** 1 The Department of Medical Genetics, Naval Medical University, Shanghai, China; 2 The Third Department of Hepatic Surgery, Eastern Hepatobiliary Surgery Hospital Affiliated to Naval Medical University, Shanghai, China

## Abstract

NAFLD is a series of liver disorders, and it has become the most prevalent hepatic disease to date. However, there are no approved and effective pharmaceuticals for NAFLD owing to a poor understanding of its pathological mechanisms. While emerging studies have demonstrated that m6A modification is highly associated with NAFLD. In this review, we summarize the general profile of NAFLD and m6A modification, and the role of m6A regulators including erasers, writers, and readers in NAFLD. Finally, we also highlight the clinical significance of m6A in NAFLD.

## INTRODUCTION

NAFLD, a spectrum of liver disorders extending from liver steatosis to NASH, is characterized by liver insulin resistance (IR) and abnormal glucose and lipid metabolism.^1^–^3^ Presently, NAFLD has become the most prevalent hepatic disease which affects ∼25% of people worldwide.^4^ However, there are no approved pharmacotherapies for NAFLD as the pathogenic mechanisms underlying NAFLD are still poorly understood so far.^5^ Therefore, it is imperative to further explore the mechanisms of NAFLD and develop novel therapeutic targets for it.

N6-methyladenosine (m6A) is one of the most abundant and essential posttranscriptional modifications in eukaryotic cells.^6^ Increasing evidence has demonstrated that m6A is involved in a variety of human diseases, comprising NAFLD,^7^ azoospermia,^8^ and heart failure,^9^ especially in human cancers.^10^–^12^ Numerous studies have recently shown that m6A plays an important role in the onset and progression of NAFLD by regulating glycolipid metabolism, IR, and chronic inflammation, implying that m6A modification may be a potential therapeutic target for NAFLD.^13^,^14^ Consequently, it is critical to investigate the mechanisms as well as the aberrant m6A modification of NAFLD to develop novel therapeutic targets and prognostic markers for NAFLD. Hence, we systematically summarized the general profile of NAFLD and m6A modification, and recent progress in understanding the roles of m6A modulators (writers, erasers, and readers) in NAFLD in this review. Finally, we also highlighted the clinical implications of m6A modification in NAFLD.

## AN OVERVIEW OF NAFLD

NAFLD is generally defined as steatosis of >5% of hepatocytes that is not caused by alcohol consumption and other specific liver damage factors.^15^ Factually, NAFLD covers a wide range of pathologies from a benign fatty liver phenotype (steatosis or excessive lipid deposition in hepatocytes) to a severe form called NASH. During the progression of NAFLD, there are a series of pathological evolutions including sustained liver inflammation, hepatocyte death, liver fibrosis, liver cirrhosis, and even liver cancer occur.^16^ It is well known that NAFLD is firmly associated with metabolic dysregulation involving de novo lipogenesis, fatty acid (FA) uptake, FA oxidation, and triglycerides (TGs) export.^17^–^19^ Given its close connection to metabolic disorders, some experts even argue that NAFLD should be replaced by metabolic-associated fatty liver disease.^20^,^21^ Nonetheless, because the definition of NAFLD cannot be completely covered by that of metabolic-associated fatty liver disease, NAFLD will be used to avoid unnecessary divergences.^22^ According to recent research, the prevalence of NAFLD is 25% to 30% in the general population and can reach 60% in obese people.^23^,^24^ It is estimated that by 2030, >300 million people in China, >100 million in the US, and 15 million to 20 million in the major European countries will have NAFLD by 2030.^25^ In the near future, NAFLD will cause enormous economic losses, but there are currently no effective targeted therapies due to a lack of understanding of its mechanisms.^26^ As a result, it is critical to investigate the mechanisms and specific pathogenesis of NAFLD to develop novel treatment strategies and improve prognosis.^27^

## AN OVERVIEW OF m6A MODIFICATION

**Figure 1:**
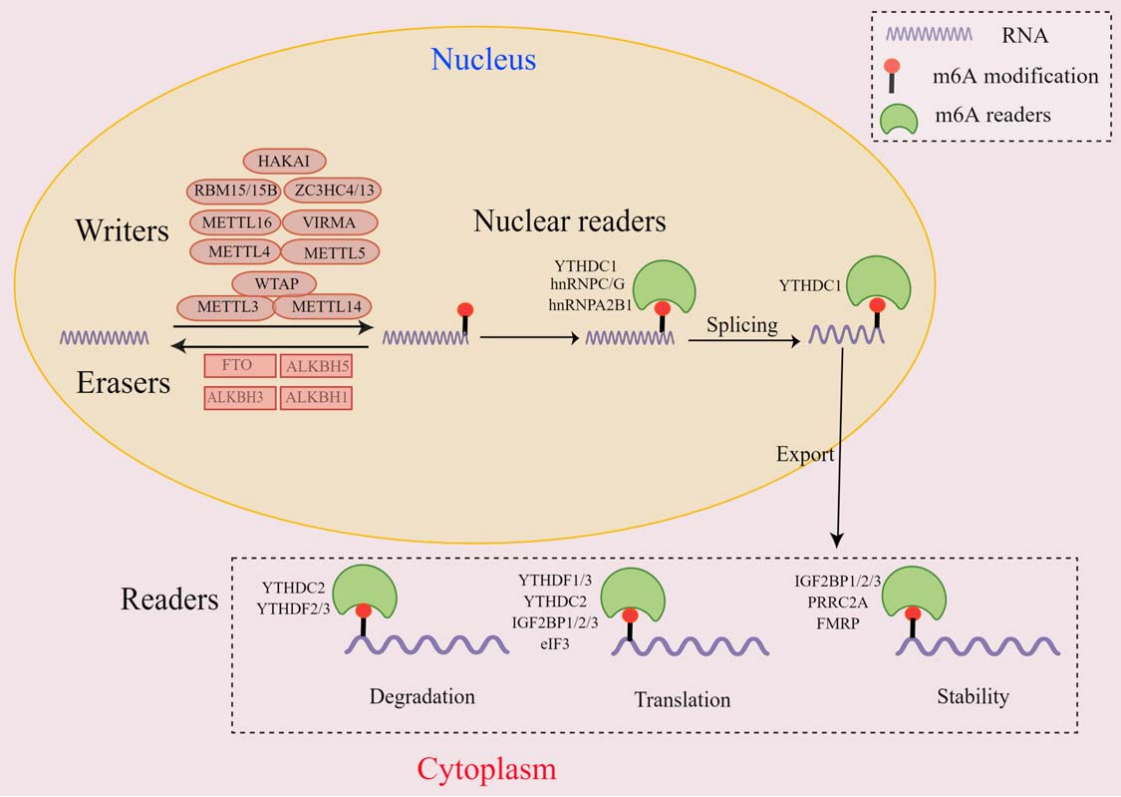
The biological process of m6A modification. m6A modification is catalyzed by methyltransferases containing METTL3, METTL5, METTL14, METTL16, WTAP, VIRMA, RBM15/15B, ZC3H13, ZC3HC4, and HAKAI. Whereas m6A modification is eliminated by demethylases containing FTO, ALKBH5, ALKBH3, and ALKBH1. In addition, m6A modification can be recognized and bound by m6A readers containing YTHDF1–3, YTHDC1–2, IGF2BP1–3, eIF3, PRRC2A, hnRNPG, hnRNPC, and hnRNPA2B1. IGF2BP1/2/3, PRRC2A, and FMRP all play a role in controlling the stability of m6A-modified RNAs. Abbreviations: ALKBH1/3/5, alkB homolog 1/3/5; eIF3, eukaryotic translation initiation factor 3; FMRP, fragile-X mental retardation protein; FTO, fat mass and obesity–associated protein; hnRNPs, heterogeneous nuclear ribonucleoproteins; IGF2BP, insulin-like growth factor 2 mRNA binding protein; m6A, N6-methyladenosine; METTL3/5/14/16, methyltransferase like-3/5/14/16; MTTP, microsomal triglyceride transfer protein; PRRC2A, proline-rich coiled-coil 2A; RBM15/15B, RNA binding motif protein 15/15B; VIRMA, Vir-like m6A methyltransferase associated; WTAP, WT1-associated protein; YTHDC1–2, YTHDF1–3,YTH N6-methyladenosine RNA binding protein 1–3; ZC3H13, zinc finger CCCH-type containing 13.

m6A modification was first identified in 1974 in poly(A) RNA fractions and has been found in a variety of eukaryotic RNAs, including mRNAs, transfer RNAs, ribosomal RNAs, circular RNAs, microRNAs, and long noncoding RNAs.^28^,^29^ In nature, the term “m6A” refers to the methyl group transfer to the N6 position of adenine, which frequently takes place at the conserved sequence DRACH (D = G/A/U, R = G/A, H = A/U/C) and enriches in stop codons, 3′ untranslated regions, and long introns.^30^–^32^ It has been reported that each mRNA in mammals contains 3 to 5 m6A modifications.^33^ The m6A modification process is dynamic and reversible, with methyltransferases (also known as “writers”) assembling, demethylases (also known as “erasers”) removing, and m6A-binding proteins (also known as “readers”) recognizing and binding (Figure 1).^34^,^35^

Increasing evidence suggests that m6A regulates the expression of target genes to influence various eukaryotic physiological and pathological processes such as self-renewal, invasion, and proliferation. It has also been well established that m6A modification influences gene expression by interfering with RNA metabolism processes such as splicing, translocation, exporting, translation, stability, and decay.^36^–^38^ Congruously, a growing amount of research indicate that dysregulated m6A modification causes aberrant metabolism of RNA related to NAFLD,^39^ which greatly promotes the development of NAFLD. Therefore, it is essential to investigate associated m6A modification to comprehend the mechanisms of NAFLD.

## ROLES OF m6A ERASERS IN NAFLD

Fat mass and obesity–associated protein (FTO), alkB homolog 5 (ALKBH5), ALKBH3, and ALKBH1 are examples of m6A erasers that are also known as demethylases.^40^–^42^ All of the demethylases listed above are members of the ALKB dioxygenase family, which consists of nonheme Fe(II)/-ketoglutarate-dependent dioxygenases.^43^ The first known demethylase, FTO, relates to NAFLD most, localizes in nuclear speckles and catalyzes the demethylation of RNA.^44^ There are 3 steps during the FTO-catalyzed demethylation process: (1) oxidase m6A to form N6-hydroxymethyladenosine (hm6A); (2) convert hm6A to N6-formadenosine (f6A); (3) convert f6A to adenosine.^45^ In addition, a recent study showed that FTO can mediate the demethylation of N6-2’O-dimethyladenosine (m6Am), which is located close to the 5′-cap of small nuclear RNA.^46^

Numerous studies have recently revealed that FTO is significantly overexpressed in the livers of NAFLD patients and animal models, indicating that FTO may play an important role in NAFLD.^47^ It has been well-documented that NAFLD is characterized by excessive lipid accumulation in the liver.^23^ Accordingly, a recent study found that overexpression of FTO reduces the m6A modification of mRNAs involved in lipid metabolism, such as fatty acid synthase, stearoyl-CoA desaturase (SCD), and monoacylglycerol O-acyltransferase 1, and thus promotes their expression, aggravating lipid accumulation in the liver.^48^ Similarly, Chen et al.^49^ found that FTO facilitates lipid accumulation in the liver by increasing nuclear translocation and maturation of sterol regulatory element-binding protein-1c (SREBP1c) and promoting the transcription of the cell death-inducing DFFA-like effector C (CIDEC). Furthermore, Hu et al.^50^ demonstrated that FTO transactivation and m6A demethylation on mRNA of lipogenic genes induced lipogenic gene activation and lipid accumulation during NAFLD and were mediated by glucocorticoid receptor. In addition, Wei et al.^4^ discovered that FTO facilitates lipid accumulation in NAFLD by suppressing the expression of the peroxisome proliferator-activated receptor α (PPARα). In addition to lipid accumulation, FTO can cause IR in the liver by increasing the expression of gluconeogenic genes such as phosphoenolpyruvate carboxykinase 1 and glucose-6-phosphatase, thereby promoting the progression of NAFLD.^51^ Further to that, overexpression of FTO reduces TG transport in the liver by decreasing the expression levels of microsomal triglyceride transfer protein, apolipoprotein B, and hepatic lipase C. This results in intracellular TG accumulation by promoting FA synthesis and inhibiting TG hydrolysis during NAFLD.^52^ To sum up, FTO promotes the development of NAFLD by modulating the m6A modification of multiple metabolism-related genes and may represent a potential therapeutic target for NAFLD.

## ROLES OF m6A WRITERS IN NAFLD

m6A writers, also referred to as m6A methyltransferases, catalyze the transfer of methyl groups from S-adenosylmethionine to the nitrogen atom (N) at the sixth position of adenine in RNA. These enzymes include methyltransferase like-3 (METTL3), METTL5, METTL14, METTL16, WT1-associated protein (WTAP), Vir-like m6A methyltransferase associated (also known as KIAA1429), RNA binding motif protein 15/15B (RBM15/15B), zinc finger CCCH-type containing 13 (ZC3H13), and HAKAI (also known as CBLL1, a RING-finger type E3 ubiquitin ligase).^45^,^53^–^56^ Among these m6A methyltransferases, METTL3 and METTL14 had the most pronounced effect on NAFLD. The core subunit of the m6A-METTL complex, METTL3, contains the sole methyltransferase catalytic domain and interacts with METTL14 to form the m6A-METTL complex–named heterodimer METTL3/METTL14. METTL14 provides functional support for METTL3 and associates with it to form m6A-METTL complex.^54^,^57^

It has been observed that overexpression of METTL3 inhibits autophagosome-lysosome fusion and hinders autophagosome degradation by targeting Rubicon mRNA to enhance its expression and, as a result, reduces hepatic lipid clearance in NAFLD.^58^ Nonetheless, Qin et al.^59^ discovered that myeloid METTL3 loss blunts the pathogenesis of NAFLD by lowering the m6A alteration of DNA damage inducible transcript 4 (DDIT4), which plays a crucial role in the regulation of macrophage activation via the reduction of mTOR and NF-κB signaling pathway activity, and raising its expression, therefore, they summarized that METTL3 played an important role in accelerating NAFLD via increasing the m6A level of DDIT4. In addition, Xie et al.^14^ demonstrated that elevated METTL3 boosted hepatic IR and stimulated lipid synthesis via N6-methylating fatty acid synthase mRNA and increasing its total mRNA level. In contrast, Li and colleagues found that hepatocyte-specific deletion of METTL3 promoted NAFL-to-NASH progression by enhancing CD36-mediated hepatic-free FA uptake and CCL2-induced inflammation, whereas hepatic upregulation of METTL3 protected against NASH progression by suppressing the expression of CD36 and CCL2. Mechanistically, METTL3 directly bound to the promoters of the *CD36* and *CCL2* genes and recruited HDAC1/2, which caused deacetylation of H3K9 and H3K27 in their promoters, consequently the transcription of *CD36* and *CCL2* was suppressed. Thus, METTL3 was identified as a previously unrecognized suppressor of the NAFL-to-NASH transition.^60^ This study revealed that the function of METTL3 in NAFLD not only in enhancing the m6A modification pathway but also in inducing histone deacetylation. Together, these findings show that METTL3’s effects on NAFLD are still controversial, and additional research is needed to understand the biological functions and mechanisms of METTL3 in NAFLD. In addition to METTL3, Yang et al.^61^ discovered that the expression of METTL14 is raised in NAFLD mice, and that METTL14 enhances de novo FA synthesis and lipid accumulation via raising the protein level of ATP citrate lyase and SCD1 by stabilizing m6A modification on their mRNA, consequently promoting the progression of NAFLD. What’s more, Qiu et al.^62^ have identified that arsenic-induced NOD-like receptor protein 3 (NLRP3) inflammasome activation contributes to hepatic IR induction during arsenic-induced NAFLD, while *NLRP3* mRNA stability was strengthened by METTL14-mediated m6A modification.

In conclusion, these data indicate that METTL3 and METTL14 play a significant role in NAFLD via influencing m6A modification. However, as there are disagreements and ambiguous aspects, additional research is required to understand the processes and therapeutical potentials of m6A methyltransferases in NAFLD.

## ROLES OF m6A READERS IN NAFLD

m6A readers are indispensable m6A-binding proteins that recognize and bind to specific RNA sequences and regulate numerous RNA life cycle activities.^63^ These m6A readers can be categorized into 3 groups based on the mechanism of m6A recognition: direct readers, m6A switch readers, and indirect readers.^35^ Direct readers are composed of eukaryotic translation initiation factor 3 (eIF3), YTH domain-containing proteins, and proline-rich coiled-coil 2A. m6A switch readers consist of heterogeneous nuclear ribonucleoproteins (hnRNPs) comprising hnRNPG, hnRNPC, and hnRNPA2B1 and insulin-like growth factor 2 mRNA binding proteins (IGF2BPs) including IGF2BP1, IGF2BP2, and IGF2BP3. And indirect reader involves fragile-X mental retardation protein.^48^,^64^,^65^ Among m6A readers, both YTH domain-containing proteins and IGF2BP2 have been shown to have an effect on NAFLD to varied degrees. YTH domain-containing proteins possess a conserved YT521-B homology domain at the C-terminus, which consists of YTHDF1–3 and YTHDC1–2.^66^ The YTH domain may identify m6A methylation and bind to m6A-modified RNA in RRACH common sequences.^67^ YTHDF1, YTHDF2, and YTHDF3 are mostly localized to the cytoplasm, whereas YTHDC1 is primarily localized to the nucleus and YTHDC2 is present in both the nucleus and cytoplasm.^48^,^65^,^68^ Functionally, YTHDF1 can attach to the m6A site near the terminal sequences and cooperate with eIF3 to increase the effectiveness of RNA translation. YTHDF2, meanwhile, regulates the degradation of m6A-dependent RNA, while YTHDF3 supports YTHDF1 and YTHDF2 in a synergistic manner.^64^,^68^,^69^ YTHDC1 can mediate the export of mRNA from the nucleus and recruit the mRNA splicing factors SRSF3 and SRSF10 to regulate RNA splicing,^70^ while YTHDC2 enhances the translational efficiency of target mRNA by binding to particular m6A sequences.^71^

YTHDC2, which binds to the mRNA of the lipogenic genes *SREBP1c*, fatty acid synthase, *SCD1*, and acetyl-CoA carboxylase 1 (*ACC1*) and thus decreases the stability and expression of these genes, has recently been shown by Zhou et al.^7^ to be strikingly downregulated in the livers of obese mice and NAFLD patients. As a result, they concluded that downregulation of YTHDC2 results in overexpression of lipogenic genes and accumulation of excessive triglycerides (TGs) in the liver in an m6A-dependent manner, which facilitates the advancement of NAFLD. In contrast, Yang et al.^61^ claimed that they had discovered an increase of eIF3G and YTHDC2 in NAFLD mice, but they did not go into detail about the underlying mechanisms and outcomes. In addition, Zhong et al.^72^ discovered that YTHDF2 binds to *PPARα* mRNA to increase its mRNA stability and expression, and then promotes lipid accumulation during hepatic steatosis caused by circadian rhythm disruption. In addition, Peng et al.^58^ demonstrated that overexpression of YTHDF1 can decrease autophagic flux and enhance lipid droplet accumulation in NAFLD by binding to *Rubicon* mRNA to boost its stability, hence accelerating the progression of NAFLD. While IGF2BP2 has been reported to drive the progression of NASH through elevating hepatic iron deposition and increasing production of hepatic-free cholesterol.^73^,^74^ In contrast, global IGF2BP2 deficiency prevents mice from NAFLD by causing resistance to obesity and fatty liver, whereas hepatocyte-specific deletion of IGF2BP2 promotes moderate diet-induced fatty liver through impairing FA oxidation by increasing mRNA degradation of *PPARα* and carnitine palmitoyltransferase 1A which were supposed to be stabilized and bound by IGF2BP2.^75^

In conclusion, these results indicate that m6A readers play a crucial and intricate role in NAFLD, although the hidden mechanisms remain poorly known and require additional research.

## CLINICAL SIGNIFICANCE OF m6A IN NAFLD

Mounting evidence confirms that m6A regulators play a vital role in the development of NAFLD; hence, it is reasonable to target these regulators to develop effective therapeutic strategies for NAFLD. Its interesting to note that a recent study found that miR-627-5p could bind to the 3′ untranslated region of FTO and may reduce IR and abnormalities of glucose and lipid metabolism in NAFLD by suppressing the production of FTO.^76^ Similarly, Lim and colleagues showed that by restoring mitochondrial function and reducing endoplasmic reticulum stress, FTO knockdown can lessen palmitic acid–induced hepatocyte lipotoxicity.^47^,^77^ Furthermore, entacapone, a possible FTO inhibitor, has been shown to be beneficial in treating metabolic disorders such as NAFLD.^78^ Meclofenamic acid, another highly selective FTO inhibitor, has also been observed to diminish the accumulation of TG in liver cells, and may help to relieve NAFLD.^79^,^80^ In addition, Chen et al.^81^ have confirmed that the methyl donor betaine can be utilized to protect against NAFLD in an FTO-dependent manner. In particular, Lu et al.^13^ demonstrated that curcumin reduces lipopolysaccharide-induced liver damage and hepatic lipid metabolism disruption in NAFLD by boosting m6A RNA methylation, and Li et al.^82^ demonstrated that exenatide ameliorates hepatic steatosis by decreasing FTO gene expression and fat mass via the PI3K signaling pathway in NAFLD. Moreover, Xie and colleagues found that METTL3 knockdown decreased *FASN* mRNA levels, which in turn inhibited lipogenesis, leading them to believe that METTL3 would be a possible therapeutic target for treating NAFLD.^14^,^59^ However, there are no effective therapies that target METTL3 to treat NAFLD currently; therefore, additional research is required to create innovative medications that target it as well as other m6A modulators. Overall, it is possible to conclude that m6A regulators are prospective therapeutic targets for NAFLD.

## CONCLUSION AND FUTURE DIRECTIONS

**Figure 2:**
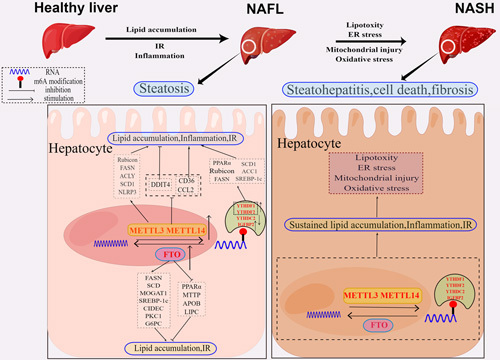
The role of m6A regulators in the development of NAFLD. In NAFLD, m6A regulators (FTO, METTL3/14, YTHDF1/2, YTHDC2) increase lipid accumulation, IR, and inflammation and decrease mitochondrial content by modulating the expression level of targeted RNAs associated with lipid metabolism including FASN, SCD, MOGAT1, SREBP1, CIDEC, PPARα, ACLY, SCD1, RUBICON, MTTP, APOB, and LIPC, and inflammation including DDIT4, CD36, CCL2, and NLRP3. In NASH, sustained lipid accumulation, inflammation, and IR caused by m6A regulators induce lipotoxicity, ER stress, mitochondrial injury, and oxidative stress, leading to steatohepatitis, cell death, and fibrosis. Abbreviations: ACC1, acetyl-CoA carboxylase 1; ACLY, ATP citrate lyase; APOB, apolipoprotein B; CIDEC, cell death-inducing DFFA-like effector; DDIT4, DNA damage inducible transcript 4; ER, endoplasmic reticulum; FASN, fatty acid synthase; FTO, fat mass and obesity–associated protein; G6PC, glucose-6-phosphatase; IGFBP2, insulin-like growth factor 2 mRNA binding protein; IR, insulin resistance; LD, lipid droplet; LIPC, hepatic lipase C; m6A, N6-methyladenosine; METTL3, methyltransferase like-3; MOGAT1, monoacylglycerol O-acyltransferase 1; MTTP, microsomal triglyceride transfer protein; NLRP3, NOD-like receptor protein 3; PCK1, phosphoenolpyruvate carboxykinase; PPARα, peroxisome proliferator-activated receptor α; SCD, stearoyl-CoA desaturase; SREBP1c, sterol regulatory element-binding protein1c; TG, triglyceride; YTHDC1–2, YTH domain-containing 1–2; YTHDF1–3,YTH N6-methyladenosine RNA binding protein 1–3.

NAFLD has gained increasing attention due to its high prevalence, lack of effective treatments, and poor clinical outcomes.^3^ While numerous studies have indicated that m6A modification modulators produce critical and complex effects in the development of NAFLD (Figure 2). Consequently, it is essential to understand these mechanisms and investigate their potential therapeutic value for NAFLD. Our present review comprehensively summarizes the profile of NAFLD and the latest understanding of the roles, mechanisms, and potential clinical significance of m6A in NAFLD. However, the specific functions and mechanisms of m6A regulators in NAFLD are complicated and still elusive. Notably, even the same regulator can exhibit opposite effects in NAFLD in different animal models. Take METTL3 as an example, Qin et al.^59^ delivered that myeloid METTL3 deficiency impedes the pathological progress of NAFLD via reducing leukocyte infiltration and hepatic damage. In contrast, Li et al.^60^ reported that hepatocyte-specific METTL3 deletion drives NAFLD-to-NASH progression by enhancing CD36-mediated hepatic-free FA uptake and CCL2-induced inflammation. Furthermore, m6A regulators may function in cooperation in NAFLD. For instance, Inhibiting autophagic flux and aggravating lipid droplet buildup in NAFLD are caused by METTL3-mediated m6A alteration, which is dependent on YTHDF1 increasing *Rubicon* mRNA stability.^58^ Collectively, the involvement of m6A regulators in NAFLD has shown complexity and diversity (Table 1), therefore, more research is needed to completely understand their functional mechanisms and clinical significance in NAFLD.

**Table 1:** Multiple functions of m6A regulators in NAFLD

Type	Regultors	Roles	Expression	Mechanisms	References
Eraser	FTO	Facilitator	Up	Increases lipid accumulation by inducing expression of FASN, SCD, and MOGAT1	48
				Increases lipid accumulation by promoting nuclear translocation, maturation of SREBP1c, and the transcription of CIDEC	49
				Increases lipid accumulation by suppressing the expression of PPARα	4
				Induce liver IR by raising hepatic gluconeogenic gene expression, containing PCK1 and G6PC	51
				Reduces TG transport in the liver by decreasing the expression levels of MTTP, APOB, and LIPC	52
Writer	METTL3	Facilitator	Up	Suppresses hepatic lipid clearance by targeting Rubicon mRNA to increase its expression	58
				Accelerates the pathogenesis of NAFLD by increasing m6A modification of DDIT4 and decreasing its expression	59
				Increase hepatic IR and promote lipid synthesis via N6-methylation of FASN mRNA	14
		Suppressor	Up	Hepatic upregulation of METTL3 protected against NASH progression by suppressing the expression of CD36 and CCL2	60
	METTL14	Facilitator	Up	Increases de novo fatty acid synthesis and lipid accumulation by enhancing the protein level of ACLY and SCD1 via stabilizing m6A modification on their mRNA	61
				Induces hepatic IR and NLRP3 inflammasome activation by mediating m6A modification of *NLRP3* mRNA during arsenic-induced NAFLD	62
Reader	YTHDC2	Suppressor	Down	Recognizes and binds to m6A-modified mRNA of *SREBP1C*, *FASN*, *SCD1*, and *ACC1*, thus regulating hepatic TG homeostasis	7
	YTHDF2	Facilitator	Up	Binds to PPARα mRNA to increase its mRNA stability and expression, afterward causes lipid accumulation	72
	YTHDF1	Facilitator	Up	Inhibits autophagic flux and increases LD accumulation via binding to *Rubicon* mRNA	58
	IGF2BP2-2	Facilitator	Up	Drives the progression of NASH through elevating hepatic iron deposition and increasing production of hepatic-free cholesterol	73,74
	IGF2BP2	Facilitator	Up	Hepatocyte-specific knockout of IGFBP2 promotes modest diet-induced fatty liver by impairing fatty acid oxidation and global IGFBP2 deficiency protects against fatty liver	75

Abbreviations: ACC1, acetyl-CoA carboxylase 1; ACLY, ATP citrate lyase; APOB, apolipoprotein B; CIDEC, cell death-inducing DFFA-like effector; DDIT4, DNA damage inducible transcript 4; FASN, fatty acid synthase; FTO, fat mass and obesity–associated protein; G6PC, glucose-6-phosphatase; IGFBP2, insulin-like growth factor 2 mRNA binding protein; IR, insulin resistance; LD, lipid droplet; LIPC, hepatic lipase C; m6A, N6-methyladenosine; METTL3, methyltransferase like-3; MOGAT1, monoacylglycerol O-acyltransferase 1; MTTP, microsomal triglyceride transfer protein; NLRP3, NOD-like receptor protein 3; PCK1, phosphoenolpyruvate carboxykinase; PPARα, peroxisome proliferator-activated receptor α; SCD, stearoyl-CoA desaturase; SREBP1c, sterol regulatory element-binding protein1c; TG, triglyceride; YTHDC1–2, YTH domain-containing 1–2; YTHDF1–3,YTH N6-methyladenosine RNA binding protein 1–3.

Fortunately, the crucial roles of m6A regulators found in NAFLD indicate that they may be promising therapeutic targets. Presently, various FTO-targeting drugs, including entacapone, meclofenamic acid, betaine, exenatide, and curcumin, have been found to be promising in the treatment of NAFLD. However, the available m6A-targeted therapies for NAFLD only target FTO; as a result, innovative therapeutics targeting additional m6A modulators should be investigated in the future. FTO is also overexpressed in NAFLD patients, which could be a sign of a poor clinical outcome, although univariate Cox regression must be done to formally recognize FTO as an independent prognostic factor for NAFLD patients.^83^ Importantly, the gene expression profile and expression level of m6A regulators, as well as clinical prognostic information for NAFLD patients, should be acquired to determine the prognostic significance of m6A regulators in NAFLD.
